# PPAR*γ* Ligand as a Promising Candidate for Colorectal Cancer Chemoprevention: A Pilot Study

**DOI:** 10.1155/2010/257835

**Published:** 2010-08-02

**Authors:** Hirokazu Takahashi, Kunihiro Hosono, Takashi Uchiyama, Michiko Sugiyama, Eiji Sakai, Hiroki Endo, Shin Maeda, Katherine L. Schaefer, Hitoshi Nakagama, Atsushi Nakajima

**Affiliations:** ^1^Gastroenterology Division, Graduate School of Medicine, Yokohama City University, 3-9 Fuku-ura, Kanazawa-ku, Yokohama, 236-0004, Japan; ^2^Gastroenterology and Hepatology Division, University of Rochester, Rochester, NY 14627, USA; ^3^Biochemistry Division, National Cancer Center Research Institute, Chuo-Ku, Tokyo 104-0045, Japan

## Abstract

Activating synthetic ligands for peroxisome proliferator-activated receptor gamma (PPAR*γ*), such as pioglitazone, are commonly used to treat persons with diabetes mellitus with improvement of insulin resistance. Several reports have clearly demonstrated that PPAR*γ* ligands could inhibit colorectal cancer cell growth and induce apoptosis. Meanwhile, aberrant crypt foci (ACF) have come to be established as a biomarker of the risk of CRC in azoxymethane-treated mice and rats. In humans, ACF can be detected using magnifying colonoscopy. Previously, CRC and adenoma were used as a target for chemopreventive agents, but it needs a long time to evaluate, however, ACF can be a surrogate marker of CRC even for a brief period. In this clinical study, we investigated the chemopreventive effect of pioglitazone on the development of human ACF as a surrogate marker of CRC. Twenty-nine patients were divided into two groups, 20 were in the endoscopically normal control group and 9 were in the pioglitazone (15 mg/day) group, and ACF and adenoma were examined before and after 1-month treatment. The number of ACF was significantly decreased (5.8 ± 1.1 to 3.3 ± 2.3) after 1 month of pioglitazone treatment, however, there was no significant change in the number of crypts/ACF or in the number and size of adenomas. Pioglitazone may have a clinical application as a cancer-preventive drug. This investigation is just a pilot study, therefore, further clinical studies are needed to show that the PPAR*γ* ligand may be a promising candidate as a chemopreventive agent for colorectal carcinogenesis.

## 1. Introduction

Peroxisome proliferator-activated receptor gamma (PPAR*γ*) is expressed in adipose tissue and plays a central role in adipocyte differentiation and insulin sensitivity. Activated synthetic ligands for PPAR*γ* are widely used as treatment for type 2 diabetes mellitus (DM) in order to improve insulin resistance. PPAR*γ* is also overexpressed in many tumors [1–5]. Several studies have reported that treatment of cancer cells with PPAR*γ* ligands induces cell differentiation and apoptosis, suggesting their potential application as chemopreventive agents against carcinogenesis [4, 6, 7]. Recent studies have suggested that PPAR*γ* has an inhibitory effect on cancer cell growth [8–10] and might inhibit cell growth and induce apoptosis in adenocarcinomas [9, 11–13], as well as affect tubulin formation *in vitro *[14]. Initial efforts have focused on activation with PPAR*γ* ligands, as these have been shown to induce G1 cell cycle arrest in a variety of tumor cell lines [15, 16]. Su et al. reported that PPAR*γ* agonist inhibits both initiation and progression of colon tumors in the AOM-mouse model study [17]. We have reported previously that PPAR*γ* ligands suppress colonic epithelial cell turnover and colon carcinogenesis through inhibition of the beta-catenin/T cell factor pathway [18], and PPAR*γ* ligands may be potential chemopreventive agents in an azoxymethane-induced colorectal carcinogenesis model and Apc^*Min* /+^ mice model [19, 20]. Colorectal cancer (CRC) is potentially one of the most preventable malignancies [21, 22]. However, the results of clinical trials with PPAR*γ* ligands in CRC have shown only modest results. This implies that focusing on PPAR*γ* as a specific antitumoral target is not likely to be successful, because PPAR*γ* ligands are not an active agent for the treatment of metastatic CRC or liposarcoma [23, 24]. Therefore, we have evaluated chemopreventive effects of PPAR*γ* ligand on the formation of the human aberrant crypt foci (ACF), which is an early stage of colorectal carcinogenesis. ACF were first discovered in mice treated with azoxymethane [25] and have become established as a biomarker of the risk of CRC in azoxymethane-treated mice and rats [26]. In humans, ACF can be detected using magnifying colonoscopy [27]. Previously, CRC and adenoma were used as a target for assessing the efficacy of potential chemopreventive agents; however, this model can only be evaluated over a long period of time. In contrast, the therapeutic efficacy of a product can be evaluated in ACF within a comparatively brief period. We report hire on the results of a study that evaluated the chemopreventive effect of PPAR*γ* ligand by using ACF as a surrogate marker of CRC.

## 2. Methods

### 2.1. Magnifying Colonoscopy for Identification of ACF

**Figure 1:**
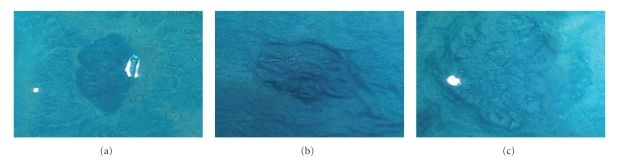
Typical features of ACF on magnifying colonoscopy with methylene blue staining.

Bowel preparation for the colonoscopy was carried out using polyethylene glycol solution. A Fujinon EC-490ZW5/M colonoscope was used to perform the magnifying colonoscopy (Fujinon Toshiba ES Systems Co., Ltd, Tokyo, Japan). Total colonoscopy was performed before imaging of rectal ACF. The exclusion criteria included: presence of contraindications to colonoscopy; current or past nonsteroidal anti-inflammatory drug use including aspirin; or family history of CRC; or history of adenoma, carcinoma, familial adenomatous polyposis, inflammatory bowel disease, or radiation colitis. Subjects with a history of colectomy, gastrectomy, or colorectal polypectomy were also excluded. Colorectal adenomas were diagnosed from pathological findings. Subsequently, 0.25% methylene blue was applied to the mucosa of the lower rectal region extending from the middle Houston's valve to the dentate line using spray catheter. ACF were described as lesions consisting of large, thick crypts in methylene blue-stained specimens of the colon (Figure 1). All ACF were recorded photographically and evaluated by two independent endoscopists who were unaware of the subjects' clinical histories. All the patients were divided into two groups, 20 were in the endoscopically normal control group and 9 were in the pioglitazone (PPAR*γ* ligand) group, (15 mg/day); ACF and adenoma were examined before and after 1 month of treatment.

### 2.2. Measurement of the Visceral and Subcutaneous Fat Areas

Body mass index (BMI) was calculated using the following equation: body weight (kg)/[height (m)^2^]. Intra-abdominal adipose tissue was assessed, as previously described, by measuring the visceral fat area (VFA), subcutaneous fat area (SFA), and waist circumference from computed tomographic (CT) images at the level of the umbilicus. All CT scans were carried out with the subjects in the supine position. The borders of the intra-abdominal cavity were outlined on the CT images, and the VFA was quantified using Fat Scan software (N2 System Corporation, Kobe, Japan).

### 2.3. Statistical Analysis

Data are expressed as mean ± standard deviation (SD), unless otherwise indicated. The relationships between the number of ACF and relevant covariates were examined by univariate regression analysis and determined using the Stat View software (SAS Institute Inc., Cary, NC, USA).

## 3. Results and Discussion

The clinical characteristics of the study participants are shown in Table 1. There were no significant differences between the groups in terms of their mean age, waist circumference, BMI, VFA, and SFA. The typical colonoscopic features of ACF are shown in Figure 1. The number of ACF was significantly decreased after 1 month's treatment with PPAR*γ* ligand compared with the controls who received no treatment (*P* = .0226), however, there was no change in the number of crypts/ACF (Table 2) and in ACF size (data not shown). Similarly, after one month of treatment there was no change in the number and size of adenoma (Table 2).

In the present study, pioglitazone treatment decreased the number of ACF, however, the number of crypts/ACF remained unchanged. These results suggest that pioglitazone affects ACF incidence rather than growth. The lack of change in the number and size of the adenomas may have been because the duration of pioglitazone administration was too short to be effective in this respect. 

The limitations of this pilot study were its small size, its short duration, and the absence of histological evaluation. Additional research in a large number of subjects is needed to elucidate the clinical effect and benefits of pioglitazone in colorectal carcinogenesis. Chemopreventive trials, the use of medications to prevent disease, have now been carried out extensively in colorectal tumors, for example, supplemental fibers [28], calcium supplementation [29], aspirin [30], nonsteroidal anti-inflammatory drugs (NSAIDs), and selective cyclooxygenase (COX)-2 inhibitors [31, 32], have all been evaluated. Higher doses and longer durations of use of NSAIDs and COX-2 inhibitors seem to be associated with greater protection from CRC and adenoma. However, these agents are associated with significant cardiovascular events and/or gastrointestinal harms [33]. Thus, the balance of benefits to risk does not favor chemoprevention by these agents in average-risk individuals. In conclusions, our preliminary results from this pilot study suggest that pioglitazone may have a preventive potential for human ACF and have a good safety profile in this patient population. Further clinical study is required to demonstrate that the PPAR*γ* ligand may be a promising candidate as a chemopreventive agent for colorectal carcinogenesis.

## Figures and Tables

**Table 1:** Clinical characteristics of study participants.

	Treatment group		
	Control	Pioglitazone	*P* value
N	20	9	
Age (years)	63.9 ± 10.0	61.8 ± 5.7	>.05
Waist Circumference (cm)	96.5 ± 13.6	91.8 ± 4.0	>.05
BMI (kg/m^2^)	24.4 ± 3.7	23.4 ± 3.1	>.05
VFA (cm^2^)	130.0 ± 61.2	146.1 ± 40.9	>.05
SFA (cm^2^)	151.5 ± 60.5	141.8 ± 53.5	>.05

Data are expressed as mean ± SD.

**Table 2:** Effect of PPAR*γ* ligand for human ACF and adenoma.

			Pre-treatment	Post-treatment	*P* value
Number of ACF	Control		5.4 ± 4.0	5.6 ± 5.8	>.05
	Pioglitazone		5.8 ± 1.1	3.3 ± 2.3	.0226

Number of Crypts/ACF	Control		16.8 ± 5.2	17.8 ± 6.4	>.05
	Pioglitazone		14.3 ± 5.9	13.6 ± 6.7	>.05

Number of adenoma	Control		2.0 ± 1.0	1.8 ± 0.8	>.05
	Pioglitazone		2.2 ± 1.5	2.3 ± 2.1	>.05

Mean size of adenoma (mm)	Control		6.9 ± 3.2	7.0 ± 3.3	>.05
	Pioglitazone		5.7 ± 2.8	5.8 ± 2.8	>.05

Location of maximum adenoma	Control	Right side	8	8	
		Left side	12	12	
	Pioglitazone	Right side	4	4	
		Left side	5	5	

Data are expressed as mean ± SD.

## Abbreviations

ACF: Aberrant crypt foci
CRC: Colorectal cancer
PPAR*γ*: Peroxisome proliferator-activated receptor gamma.
